# Outcome of mock embryo transfer before the first IVF cycle: A randomized control trial

**DOI:** 10.18502/ijrm.v13i11.7962

**Published:** 2020-11-22

**Authors:** Amol Borkar, Amit Shah, Anil Gudi, Roy Homburg

**Affiliations:** Fertility Unit, Homerton University Hospital, London, UK.

**Keywords:** IVF, Mock embryo transfer, Pregnancy outcomes, Live birth.

## Abstract

**Background:**

There is a lack of agreement among fertility specialists with regard to the routine use of mock embryo transfer (MET) before each in vitro fertilization (IVF) treatment cycle. While MET may be beneficial with previous difficult embryo transfer cases, its routine use before first IVF cycle has not been evaluated.

**Objective:**

To find out the effect of MET before the first IVF cycle on clinical pregnancy rate.

**Materials and Methods:**

This is a single-centre randomized controlled trial with a balanced randomization (1:1), carried out between November 2015 and October 2017, with 200 subjects at Homerton university hospital, London, randomized into either MET or control. The primary outcome was clinical pregnancy rate (detection of heart activity on the ultrasound scan), the secondary outcome measures were live birth rate, miscarriage and multiple pregnancy rates, difficult ETs, rate of blood or mucus on the catheter tip.

**Results:**

No significant differences were observed in the baseline or cycle characteristics between the two groups. The clinical pregnancy rate was similar between the MET and control groups based on both intension to treat and per protocol analyses (p = 0.98, p = 0.92, respectively). Additionally, no significant difference was seen in the live birth rate in both groups on intension to treat and per protocol analyses (p = 0.67, p = 0.47), respectively.

**Conclusion:**

Our study concludes that MET prior to first IVF cycle may not improve the success rate in young women without risk factors for a difficult embryo transfer.

## 1. Introduction

The advent of assisted reproductive technology (ART) has given some hope to several women and couples who previously had no hope of achieving pregnancies. ART involves several sequences of procedures. The embryo transfer (ET) procedure is a vitally important part of the in vitro fertilisation/ intracytoplasmic sperm injection (IVF/ICSI) process. Multiple factors like quality of embryos, technique of transfer, receptivity of endometrium can affect pregnancy outcome after ET (1). The importance of correct ET technique for successful IVF cannot be overemphasized as ET is viewed as the most crucial and critical step in an assisted conception treatment cycle. Poor ET technique may be responsible for up to 30% failure rates in ART (2-4).

There are several suggestions for overcoming the mechanical problems of ET technique. These include proper evaluation of the length and the direction of the uterine cavity to discover any unanticipated difficulty in entering the uterine cavity while performing real ET and also to choose the most suitable catheter for ET (2). MET was therefore introduced to minimize a potential mechanical problem encountered at ET and to improve the pregnancy rate (5). MET and ultrasound can be used for evaluation of uterine cavity. The details of uterocervical angulation and length may make ET easy. Studies have shown that ultrasound guided transfers are better in term of clinical pregnancy outcomes and ongoing pregnancy rates compared to clinical touch method (6-8).

There is a trend to perform this procedure as a routine for all-comers before the first cycle of IVF treatment even in the absence of any risk factors. In addition recently MET has been very popular in private practice, evidence to offer such treatments are questionable. There is therefore an obvious need for a study evaluating MET in women with low risk factors for difficult transfer in order to determine whether it should be adopted as a routine procedure. We performed a randomized controlled trial to examine the effectiveness of MET for women undergoing their first cycle of IVF without any risk factors suggestive of potentially difficult ET.

## 2. Materials and Methods

This is a single-centre randomized controlled trial with a balanced randomization (1:1), conducted at a tertiary referral unit in London, UK. The inclusion criteria of the study were couples with primary subfertility undergoing their first cycle of IVF or ICSI, where the female partner was aged between 23 and 42 completed years, with a body mass index (BMI) of 18-35, in a fresh or frozen cycle. The exclusion criteria, on the other hand, were known uterine anomaly, previous cervical surgery, cone biopsy, large loop excision of the transformation zone, previous ET or hysteroscopy.

Eligible couples were randomiszed to the intervention of MET with a soft ET catheter (Wallace) on an average three months before the actual ET. The findings of MET or non-intervention were noted on a study proforma to guide the actual ET. A soft Wallace catheter was used to sound the uterus without ultrasound guidance and to measure utero-cervical length (UCL) and in addition to note the position of the uterus, external cervical os appearance, easy or difficult procedure. The uterine cavity length at MET was recorded as the distance in centimeters from the external cervical os to the uterine fundus. Both groups had 1 or 2 top-quality embryos transferred either on day 5 blastocyst or day 3 cells stage.

All subjects were followed until the end of the time horizon. Those who opted out from the trial and didn't proceed to an actual ET were noted. All randomized couples were analyzed in their allocated group as per ITT analysis as well as analysis per protocol/ ET. While the primary outcome was clinical pregnancy rate (detection of heart activity on the ultrasound scan), the secondary outcome measures were live birth rate, miscarriage and multiple pregnancy rates, difficult ETs, rate of blood or mucus on the catheter tip.

Computer randomized numbers were utilized. Consecutively numbered, individual opaque envelops were used for allocation. Eligible subjects were given verbal information as well as written information about the trial. At the next appointment the consent form was signed followed by opening of the consecutively numbered sealed envelope by the research coordinator to assign the patient for the intervention. The majority of the women in the study underwent a frozen embryo replacement cycle. These women had mock transfer immediately after oocyte retrieval and actual ET was carried out in three months following the oocyte retrieval as this was the waiting time for frozen embryo replacement cycle. Subjects who underwent fresh ET had mock transfer about three months before the start of the fresh IVF cycle. After the intervention mock, ET findings were noted in the proforma which was filed in the notes with an aim to review during actual embryos transfer process. Women who had a difficult MET were offered either a senior consultant review or hysteroscopy and cervical dilatation before the actual ET. The doctor (AB) performing MET was also trained in performing ETs as per standard unit policy. Due to the intervention involved in the trial, blinding was not possible. This was unlikely to affect the outcome of the trial, as the outcome was objective.

### Ethical consideration

The study has been approved by the local ethics committee at Homerton university hospital (REC reference 13/LO/1834). All the participants gave their informed consent.

### Statistical analysis

Based on the ITT and per protocol principles, statistical comparisons were carried out using two sample *t* tests and epidemiological aspects to calculate relative risk with its significance value and confidence intervals. Also, we used more informative summary statistics where appropriate with the Statistical Program for Social Sciences (SPSS, Inc., Version 21.0, Chicago, IL, USA). A two-sided P-value, 0.05 was considered as statistically significant.

## 3. Results

**Figure 1:**
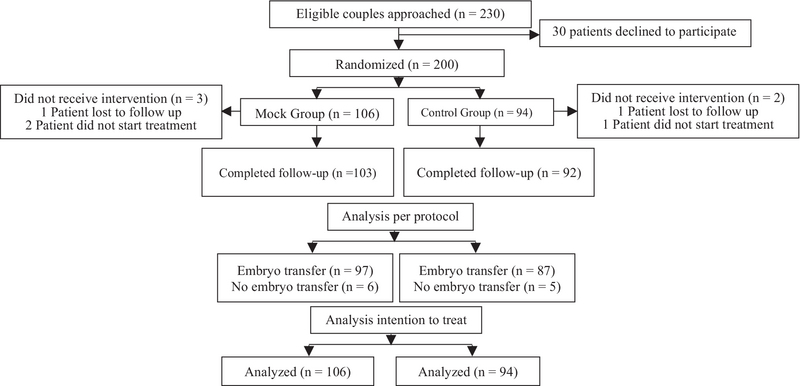
Consort flowchart.

Between November 2015 and October 2017, 230 couples undergoing their first IVF/ICSI cycle were approached. Of these, 200 were randomized to either mock ET or control groups (Figure 1). There were no differences between the two groups in terms of baseline characteristics (Table I). Shapiro-Wilk Normality test was used to check normality.

### Mock ET group

Of the 106 subjects randomized to the Mock ET group, 9 did not reach the stage of ET, and two never started their treatment cycle. Besides, one patient was lost to follow-up, while six subjects had stimulation for the IVF/ICSI cycle but didn't undergo ET either because of personal reasons, a spontaneous conception, or there was no embryo available due to failure of fertilization. MET findings showed the average UCL was 7.4 in our study population. Twenty-three of the ninety-six (23.95%) subjects had a pin-point cervix at the time of MET and one had fibrosis at the external cervical os. A total of 97 of the 106 subjects in the mock group had ET. Of these, 93 (95.87 %) subjects had an easy mock transfer with soft ET catheter (Wallace), while 3 met difficulty in the MET with the soft catheter, where in MET was performed easily with routine ET catheter using a stylet. The actual ET for all of the three cases experienced no difficulty in the transfer. However, only one patient (1.03%) had a difficult mock transfer even with the use of a stylet and she underwent hysteroscopy and cervical dilatation before the actual ET. All the METs were performed by registrars except one that was performed by a consultant. Accordingly, 60 out of the 97 (61.85 %) actual ETs were performed by registrars and 37 (38.14%) by the consultants. All ETs were done with the Wallace Sure Pro catheter as a standard practice in our unit. Only one patient (1.03%) had difficulty with actual ET in spite of easy MET as per the operators notes but there was no evidence of bleeding or mucus at the tip of the catheter. All the rest of the subjects had an easy ET as per the documentation in the notes.

In total, 25 out of 97 (25.77%) subjects had retroversion of the uterus, 3 (3%) had mid-position or axial uterus, and 68 (70.1%) had an ante-verted uterus. In one subject, it was not possible to find out the position due to difficulty in MET. Four out of ninety-seven (4.12%) subjects' ET findings revealed blood at the tip of catheter, and in seven (7.21%), cases there was mucus at the tip of the catheter. In addition, 85 of the 97 (87.62%) MET group subjects had either one or two top-quality embryos transferred. The average number of embryos transferred in the MET group was 1.26. In the MET group, 71 of the 103 (68.93%) subjects had single and 26 (25.24%) had double ETs. None underwent triple ET in the MET group; 88% of these subjects had day 5 embryos transferred. Of the 97 subjects, 47 (48.45%) had a positive HCG test and 31 (31.95%) had a live birth; 10 of the 47 (21.27%) subjects had miscarriage, 4 (8.15%) biochemical, 1 (2.12%) ectopic, and 1 (2.12%) had termination of pregnancy for congenital abnormality.

### Control group

Of the 94 subjects randomized to the control group, 7 did not reach the stage of ET. One patient didn't start the treatment cycle and one was lost to follow-up. Five subjects had stimulation for the IVF/ICSI cycle but didn't undergo ET either due to personal reasons, a spontaneous conception, or absence of embryo due to failure of fertilization. A total of 87 subjects in the control group had actual ET. All ETs were done with the Wallace Sure Pro catheter as a standard practice in our unit (Figure 1). None of these subjects had any difficulty during the ET. Four out of the 87 (4.59%) patient's ET findings revealed blood at the tip of catheter and in 6 (6.89%) cases there was mucus at the tip of the catheter.

Out of the 87 subjects (87.35%), 76 in the control group had either one or two top-quality embryos transferred. The average number of embryos transferred in the control group was 1.32. While 60 out of 92 (65.21%) had single ET in the control group, 26 (31.52%) had double ET. Only one patient (1.08%) underwent triple ET due to her age and average quality of the embryo. In the control group, 85% subjects had day 5 embryos transferred. Of the 87, 51 (58.62%) ETs were performed by registrars and 36 (41.37%) by the consultants. A total of 38 (43.67%) subjects had a positive HCG, while 25 (28.73%) had a live birth and 13 (14.94%) had miscarriage. As presented in Table II, no significant difference were seen between the two groups in terms of protocol used, IVF/ICSI procedure, fresh or frozen ET, quality and number of embryos transferred,. Although the number of oocytes was different between the groups (p = 0.03), we do not believe this could have influenced the outcomes of our study question (Table II). The statistical test used is two sample *t* test.

### Primary outcomes 

There was no significant difference in the clinical pregnancy rates between the MET and control groups based on both ITT [40.56% (43/106) vs 40.42% (38/94); RR 1.0035 (95% CI = 0.7167 - 1.4051), p = 0.98] and per protocol [44.32% (43/97) vs 43.67% (38/87); RR 1.0149 (95% CI = 0.7321-1.4069), p = 0.92] analyses (Table III).

### Secondary outcomes

There was no significant difference in the live birth rate between the MET and control groups based on ITT [29.24% (31/106) vs 26.59% (25/94); RR 1.0996 (95% CI = 0.7027-1.7207), p = 0.67] and per protocol [31.95% (31/97) vs 28.73% (25/87); RR 1.1122 (95% CI = 0.7161-1.7273), p = 0.47] analyses. In addition, there were no significant differences in the miscarriage nor multiple pregnancy rates based on both the ITT and per protocol analyses (Table III). The statistical test used is two sample *t* test. Most importantly in the control group, none of the subjects had difficult ET, while in the MET group only one patient had difficult ET. The MET group identified four difficult transfers, of which only one required intervention of hysteroscopy and cervical dilatation. Both the groups had similar incidence for the finding of catheter tip bleeding and/or mucus which can be a sign of difficult ET. The doctors performing actual ET were qualified as per units standard practice measures. There was no statistically significant difference in the two different operators performing the actual transfers in both the groups.

**Table 1:** Baseline Characteristics of the Mock and control group


**Variables**	**Mock (mean)**	**Control (mean)**	**P-value**	**Min**	**Max**	**Range**
**Age (yr)**	32.00	33.00	0.06	24.00	42.00	18.00
**Duration of subfertility (months)**	39.89	39.84	0.99	12.00	120.00	108.00
**BMI (kg/m ${}^{2}$)**	24.703	24.690	0.97	18.40	35.00	16.60
**AFC (n)**	26.87	24.99	0.32	5.00	75.00	70.00
**FSH (IU/L)**	5.208	5.510	0.17	1.00	11.90	10.90
**AMH (pmol/l)**	35.4000	30.9400	0.63	3.20	121.55	118.35
Shapiro-Wilk Normality test and Student *t* test, BMI: Body mass index, AMH: Anti-Müllerian hormone, FSH: Follicle-stimulating hormone; AFC: Antral follicle count						

**Table 2:** Cycle characteristics of the mock and control groups


	**MOCK (n = 103)**	**Control (n = 92)**	**P-value**	**Relative risk (95% CI)**
**Protocol**					
	**Long agonist **	4/103 (3.88)	01/92 (1.09)	0.25	3.5728 (0.4066-31.3931)
	**Antagonist **	99/103 (96.12)	91/92 (98.91)	0.20	0.9717 (0.9296-1.0158
**Insemination**					
	**IVF **	47/103 (45.63)	37/92 (40.22)	0.44	1.1346 (0.8187-1.5725)
	**ICSI **	32/103 (31.07)	37/92 (40.22)	0.18	0.7725 (0.5280-1.1302)
	**Split**	24/103 (23.30)	18/92 (19.57)	0.52	1.1909 ( 0.6922-2.0490)
	**Fresh **	5//97 (4.85)	09/87 (10.34)	0.16	0.4693 (0.1633-1.3481)
	**Frozen**	92/97 (89.32)	78/87 (89.66)	0.94	0.9963 (0.9035-1.0986)
Number of oocytes obtained*		18 (5-64)	15 (3-41)	0.03	
**Quality of embryos**					
	**Top**	85/97 (91.75)	76/87 (87.36)	0.95	1.0031 (0.8991-1.1192)
	**Average**	12/97 (12.37)	11/87 (12.64)	0.95	0.9784 (0.4552-2.1032)
**Number of transferred embryos**					
	**0**	6/103 (5.83)	05/92 (5.43)	0.90	1.0718 (0.3384-3.3954)
	**1**	71/103 (68.93)	60/92 (65.22)	0.58	1.0570 (0.8674-1.2880)
	**2**	26/103 (25.24)	26/92 (28.26)	0.63	0.8932 (0.5609-1.4223)
	**3**	0/103 (0 )	01/92 (1.09)	0.45	0.2981 (0.0123-7.2284)
**Actual transfer**					
	**Easy **	96/97 (98.97)	87/87 (100)	0.31	0.9897 (0.9698-1.0100)
	**Difficult **	01/97 (1.03)	0/87 (0)	0.54	2.6939 (0.1112-65.2795)
**Catheter tip bleeding**		04/97 (4.12)	04/87 (4.60)	0.87	0.8969 (0.2313-3.4784)
**Catheter tip mucus**		07/97 (7.22)	06/87 (6.90)	0.93	1.0464 (0.3657-2.9942)
**Consultant**		37/97 (38.14)	36/87 (41.38)	0.65	0.9218 (0.6457-1.3161)
**Registrar**		60/97 (61.86)	51/87 (58.62)	0.65	1.0552 (0.8335-1.3358)
Data presented as n (%). P-value refer to Chi-squared test; when appropriate, P-value < 0.05 was considered as significant. * independent *t* test, IVF: In vitro fertilisation, ICSI: Intracytoplasmic sperm injection					

**Table 3:** Pregnancy outcomes mock and control groups (ITT and per protocol analyses)


**Analysis by ITT (n = 200)**	**MOCK (n = 106)**	**Control (n = 94)**	**Relative risk (95% CI)**	**P-value**
**Clinical pregnancy rate**	43/106 (40.56)	38/94 (40.42)	1.0035 (0.7167-1.4051)	0.98
**Live birth rate **	31/106 (29.24)	25/94 (26.59)	1.0996 (0.7027-1.7207)	0.67
**Miscarriage rate **	10/47 (21.27)	13/38 (34.21)	0.6219 (0.3073-1.2585)	0.18
**Multiple pregnancy rate **	3/43 (6.97)	3/38 (7.89)	0.8837 (0.1895-4.1209)	0.87
**Analysis per protocol /ET (n = 184)**	**Mock (n = 97)**	**Control (n = 87 )**	**Relative risk (95% CI)**	**P-value**
**Clinical pregnancy rate**	43/97 (44.32)	38/87 (43.67)	1.0149 (0.7321-1.4069)	0.92
**Live birth rate **	31/97 (31.95)	25/87 (28.73)	1.1122 (0.7161-1.7273)	0.47
**Miscarriage rate **	10/47 (21.27)	13/38 (34.21)	0.6219 (0.3073-1.2585)	0.18
**Multiple pregnancy rate **	3/43 (6.97)	3/38 (7.89)	0.8837 (0.1895-4.1209)	0.87
Data presented as n (%), ITT: Intension to treat, Chi-squared test, P-value < 0.05 was considered as significant. Clinical pregnancy: Detection of heart activity on the ultrasound scan; Live birth rate: The percentage of all cycles that lead to live birth (more than 20 wk)				

## 4. Discussion

From the present study, MET before the actual ET did not result in significant improvement in the clinical pregnancy rates or live birth rates among women undergoing their first IVF/ICSI cycle with no evidence of potential risk factors anticipating difficult ET. It has been suggested that one of the reasons for performing a Mock ET is to aid assessment of uterine size and position. However, the uterine findings at a mock ET are not always a definite predictor of uterine length and position at the time of the real ET. The uterine length and position is known to be variable due to the effect of hormones on the uterine muscle and on the size of the ovaries. To minimize this variation in the uterine length, the majority of ETs were done at the time of oocyte retrieval where there is an influence of hormones and actual transfer was also performed in the hormone medicated frozen cycle (9-11).

Similarly, in our study, MET was performed at the time of oocyte retrieval, where the hormonal effect would be present and actual ET was also performed in a hormone-medicated frozen ET cycle. Uterine depth is very much different with pre-cycle blind MET compared to ultrasound guided measurement at the ET. Hence it is not the best predictor for assessing final ET depth (10). Although in our study the UCL was measured during MET, the actual transfer was performed as per unit's policy of transferring the embryo in the lower 2/3 rd of the uterine cavity under trans-abdominal ultrasound guidance. The uterine position at a mock ET cannot also always be a true predictor of the position of the uterus at the time of a real ET as uterine position can vary (9). Henne (2004) conducted a study to determine the consistency in the uterine position between mock and real ET. Henne and coworker reviewed the 996 consecutive ET cycles (585 subjects); 74% of subjects had an anteverted (AV) uterus and 26% had a retroverted (RV) uterus at MET. In our study, the results very much resemble the aforementioned study. Although the primary outcome seems obvious, a larger sample would be needed to confirm live birth outcome.

There are variable opinions from different practitioners about the exact timings of Mock ET. It has variously been performed either prior to initiation of IVF/ICSI treatment cycle or immediately before the real ET (5, 12-14). In our study, we deliberately removed the positive as well as negative effect of endometrial injury during MET at the time of oocyte retrieval by deferring actual ET for three months or more in a frozen cycle as majority of our subjects were hyper-responders (Table II) and underwent a freeze all cycle as per the units protocol to minimize risk of ovarian hyper stimulation. Difficult ET and its effect on clinical pregnancy outcome is not very clear. In the literature, some studies have shown that there is a correlation between difficult ET and reduced pregnancy rate (15, 16). On the other hand some studies have suggested that difficult or repeated ET does not adversely affect the outcome of IVF (17). Mansour and colleagues in a randomized trial showed that lower pregnancy rate in the controls is due to the very low pregnancy rate (4%) in difficult ET cases. Dummy ET can avoid unexpected difficult and failed ET (5). This is in contrast to our study where the pregnancy rates are not statistically different between the two groups. The major difference in these two studies is the use of ultrasound guidance during ET which was not used in the earlier studies making the success rates variable. Although choosing the type of catheter may be a benefit of MET, in our study we had three cases where we used a different type of catheter for MET, as not all catheters are suitable for every patient. MET is very helpful in difficult subjects and for selecting the most suitable catheter. Sometimes it is difficult to achieve proper straight alignment between the cervix and uterus due to acutely tilted utero-cervical angle. Proper evaluation of these cases can be done just before loading the embryos or in pre-treatment MET procedures. Full bladder can also help in straightening the utero-cervical angle.

A study by Tomas and co-workers concluded that difficult ET markedly reduces the pregnancy rate. Need for cervical dilatation or catheter change or blood on the catheter indicates difficult ET. Easy or intermediate transfers resulted in a 1.7-fold higher pregnancy rate than difficult transfers (18). In our study, 24 women had a pin-point cervix at the time of MET but none of them had any difficulty either during METs or actual ETs. This suggests that pin-point cervix can be a common finding (23.29% in our study) in the nulliparous woman and should not be a sole indication for MET. Several studies have shown that blood and/or mucus on the tip of catheter may indicate difficult transfer and can affect the pregnancy outcome (1, 19-21). In this study, we did not find any difference between the occurrence of blood or mucus at the tip of catheter in both the groups. Currently, training in medicine is competency based. In ART, performance of mock ET is likely to have a positive impact on training. Mock ET can be a practice procedure to improve on competence of trainees before they are deemed competent to perform the actual procedure. In a survey by Wittenberger and colleague recommended that hands-on approach should be included for ET training in reproductive endocrinology and infertility fellowship training (22).

## 5. Conclusion

Our study concludes that MET prior to first IVF/ICSI cycle may not improve the success rates in young women without risk factors for a difficult ET. MET doesn't improve the pregnancy rate and difficult ETs are not very common. In addition, MET procedure will require additional resources including catheter costs, staff availability, appointments, and extra visits for the subjects. MET should be reserved for subjects with risk factors for potential difficult ET.

##  Conflict of Interest

The authors declare that there is no conflict of interest.
